# Reply to Manzar, S. Comment on “Brinkis et al. Nutrient Intake with Early Progressive Enteral Feeding and Growth of Very Low-Birth-Weight Newborns. *Nutrients* 2022, *14*, 1181”

**DOI:** 10.3390/nu14132653

**Published:** 2022-06-27

**Authors:** Rasa Brinkis, Kerstin Albertsson-Wikland, Rasa Tamelienė, Asta Vinskaitė, Kastytis Šmigelskas, Rasa Verkauskienė

**Affiliations:** 1 Department of Neonatology, Lithuanian University of Health Sciences, 44307 Kaunas, Lithuania; rasa.tameliene@lsmuni.lt (R.T.); vinskaite@gmail.com (A.V.); 2 Department of Physiology/Endocrinology, Institute of Neuroscience and Physiology, Sahlgrenska Academy, University of Gothenburg, 40530 Gothenburg, Sweden; kerstin.albertsson.wikland@gu.se; 3 Health Research Institute, Faculty of Public Health, Lithuanian University of Health Sciences, 44307 Kaunas, Lithuania; kastytis.smigelskas@lsmuni.lt; 4 Institute of Endocrinology, Lithuanian University of Health Sciences, 44307 Kaunas, Lithuania; rasa.verkauskiene@lsmuni.lt

We appreciate Dr. Shabih Manzar’s interest [1] in our recently published work, “Nutrient Intake with Early Progressive Enteral Feeding and Growth of Very Low-Birth-Weight Newborns” [2] and his comments on several issues in our study.

*Rate of necrotizing enterocolitis*

**Figure 1:**
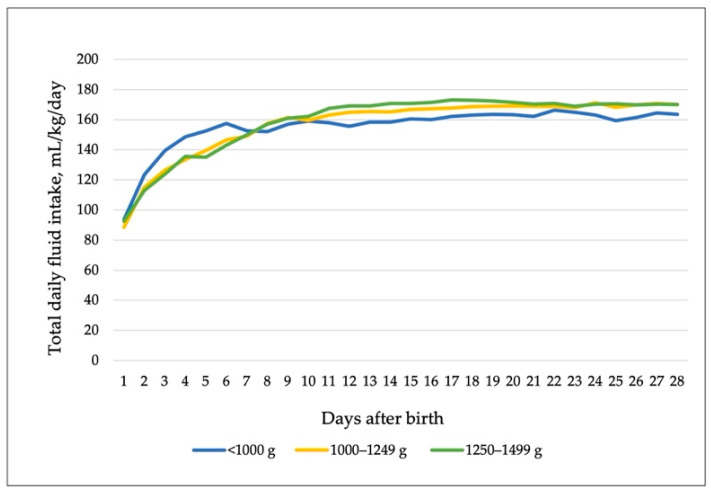
Total daily fluid (parenteral and enteral) intake in different birth-weight groups. Data are medians.

We included 120 infants in the initial cohort and did not exclude, but censored, their data on the day of death or bowel resection, which is stated in the flowchart (Figure 1) [2]. The aim of the study was to assess nutrient intake and its relationship with early growth; thus, we assumed that infants with partial bowel resection would have some degree of malabsorption, and their growth should not be compared to those with an intact bowel. We excluded these infants’ data from the nutritional and growth analyses, but we used the demographic data of the initial cohort in Table 1 [2]. Thus, we reassure the reader that the total number of infants with necrotizing enterocolitis and spontaneous intestinal perforation was eight in the whole cohort, six of whom were in the <1000 g birth-weight group.

*Fluid intake and patent ductus arteriosus (PDA)*

Daily fluid intake in Figure 3e [2] is separated to parenteral and enteral intake, and it does not exactly represent total daily intake. Our aim was to describe the trends of enteral and parenteral fluid intakes in the real picture of such an early transition phase. In our article we discussed the challenge of balancing between fluid overload and nutrient intake, though with a greater focus on potential nutrient deficit. More detailed data on total daily fluid intake in different birth-weight groups is presented in Table 1 [2], which indicates higher intake in the smallest infants during the first week, but lower intake afterwards.

There is no clear definition regarding the exact volume of parenteral or enteral fluid that may contribute to a higher risk of PDA, but we agree with the comment that it is important to be aware of the total daily fluid given during the transition from parenteral to enteral nutrition, especially in early life. The incidence of hemodynamically significant PDA in our cohort was 65.2% in the <1000 g group, similar to the rates reported in [3]; 23.2% in 1000–1249 g group; and 8.8% in 1250–1499 g group.

*Growth*

Adding the calculations with the author’s suggested triple method, the median weight-gain velocity from birth to discharge, calculated using the 2-point method, was 13.9, 13.7, and 12.9 g/kg/day in birth-weight groups G1 (<1000 g), G2 (1000–1249 g), and G3 (1250–1499 g), respectively. Weight-gain velocity calculated using the exponential method was 15.6, 14.6, and 13.2 g/kg/day in G1, G2, and G3, respectively. The weight-gain ratio (WGR) was 0.93 in G1, 0.87 in G2, and 0.78 in G3, indicating that the weight-gain ratio of the smallest newborns was closer to the suggested values, as was the change in weight z-scores that we provided in our article. However, this triple method includes only the weight gain. We strongly support the approach that linear growth, i.e., length and head circumference, should be assessed concurrently [4]. Body length reflects lean body-mass accretion, and head-circumference growth reflects the brain growth, both contributing to optimal body composition and neurodevelopmental outcomes [5,6,7].
